# Early-onset primary antibody deficiency resembling common variable immunodeficiency challenges the diagnosis of Wiedeman-Steiner and Roifman syndromes

**DOI:** 10.1038/s41598-017-02434-4

**Published:** 2017-06-16

**Authors:** Delfien J. Bogaert, Melissa Dullaers, Hye Sun Kuehn, Bart P. Leroy, Julie E. Niemela, Hans De Wilde, Sarah De Schryver, Marieke De Bruyne, Frauke Coppieters, Bart N. Lambrecht, Frans De Baets, Sergio D. Rosenzweig, Elfride De Baere, Filomeen Haerynck

**Affiliations:** 1 Clinical Immunology Research Lab, Department of Pulmonary Medicine, Centre for Primary Immunodeficiency, Jeffrey Modell Diagnosis and Research Centre, Ghent University Hospital, Ghent, Belgium; 2 Department of Paediatric Immunology and Pulmonology, Centre for Primary Immunodeficiency, Jeffrey Modell Diagnosis and Research Centre, Ghent University Hospital, Ghent, Belgium; 3 Center for Medical Genetics, Ghent University and Ghent University Hospital, Ghent, Belgium; 4 Laboratory of Immunoregulation, VIB Inflammation Research Centre, Ghent, Belgium; 5 Department of Internal Medicine, Ghent University, Ghent, Belgium; 6 Immunology Service, Department of Laboratory Medicine, NIH Clinical Centre, National Institutes of Health, Bethesda, MD USA; 7 Department of Ophthalmology, Ghent University Hospital, Ghent, Belgium; 8 Division of Ophthalmology, The Children’s Hospital of Philadelphia, Philadelphia, USA; 9 Department of Paediatric Cardiology, Ghent University Hospital, Ghent, Belgium; 10 Department of Paediatric Allergy and Immunology, Montreal Children’s Hospital, Montreal, QC Canada; 11 Department of Pulmonology, Ghent University Hospital, Ghent, Belgium

## Abstract

Syndromic primary immunodeficiencies are rare genetic disorders that affect both the immune system and other organ systems. More often, the immune defect is not the major clinical problem and is sometimes only recognized after a diagnosis has been made based on extra-immunological abnormalities. Here, we report two sibling pairs with syndromic primary immunodeficiencies that exceptionally presented with a phenotype resembling early-onset common variable immunodeficiency, while extra-immunological characteristics were not apparent at that time. Additional features not typically associated with common variable immunodeficiency were diagnosed only later, including skeletal and organ anomalies and mild facial dysmorphism. Whole exome sequencing revealed *KMT2A*-associated Wiedemann-Steiner syndrome in one sibling pair and their mother. In the other sibling pair, targeted testing of the known disease gene for Roifman syndrome (*RNU4ATAC*) provided a definite diagnosis. With this study, we underline the importance of an early-stage and thorough genetic assessment in paediatric patients with a common variable immunodeficiency phenotype, to establish a conclusive diagnosis and guide patient management. In addition, this study extends the mutational and immunophenotypical spectrum of Wiedemann-Steiner and Roifman syndromes and highlights potential directions for future pathophysiological research.

## Introduction

Common variable immunodeficiency (CVID) is one of the most frequently diagnosed primary immunodeficiencies (PIDs), and is defined as decreased serum immunoglobulin (Ig) G, decreased IgA and/or IgM, poor antibody responses to vaccines, and exclusion of other causes of hypogammaglobulinemia^1^. Patients commonly experience recurrent (sinopulmonary) infections and features of immune dysregulation such as autoimmunity^1, 2^. About 25% of CVID patients are diagnosed in childhood^3^. To rule out transient hypogammaglobulinemia of infancy, in which Ig levels spontaneously resolve mostly by the age of two to four years, a definite diagnosis of CVID should not be given before the age of four years^1^.

Here, we report novel familial cases of Wiedemann-Steiner syndrome (WSS) and Roifman syndrome (RS) that were initially categorized as early-onset CVID. WSS and RS are rare syndromic PIDs, affecting the immune system as well as other organ systems^4–6^. Although there is considerable phenotypic heterogeneity in both syndromes, hallmark extra-immunological features are generally evident very early in life^7, 8^. WSS is typically characterized by *hypertrichosis cubiti*, growth retardation, developmental delay and facial dysmorphism, and is caused by heterozygous mutations in *lysine methyltransferase 2* *A* (*KMT2A*)^7, 9–14^. *KMT2A* (also called *mixed-lineage leukemia*, *MLL*) encodes a histone methyltransferase involved in regulating chromatin-mediated transcription and is a frequent target of chromosomal rearrangements in childhood leukemia^9, 15^. WSS has only been recently associated with primary antibody deficiency^7^. RS, on the other hand, is commonly featured by antibody deficiency as well as growth retardation, spondyloepiphyseal dysplasia and retinal dystrophy^8, 16^. Biallelic mutations in *RNU4ATAC*, a noncoding small nuclear RNA (snRNA) gene, were recently identified as a cause of RS^8^. U4atac snRNA is an important component of the minor spliceosome required for minor intron splicing^8^.

This report aspires to increase awareness among immunologists and geneticists that a CVID phenotype can be the principal presentation of WSS and RS in early childhood, which is exceptional and has not been previously reported. The prior diagnosis of early-onset CVID diverted attention away from the initially less evident extra-immunological features, which significantly delayed identification of the underlying syndromic disorders. Additionally, in both families we identified mutations that have not been previously associated with disease. We aimed to provide insight as to how these mutations are disease-causing. Finally, we expand the immunophenotypical spectrum of WSS and RS, which could support future mechanistic research.

## Results

### An early-onset CVID phenotype in two unrelated sibling pairs

**Figure 1:**
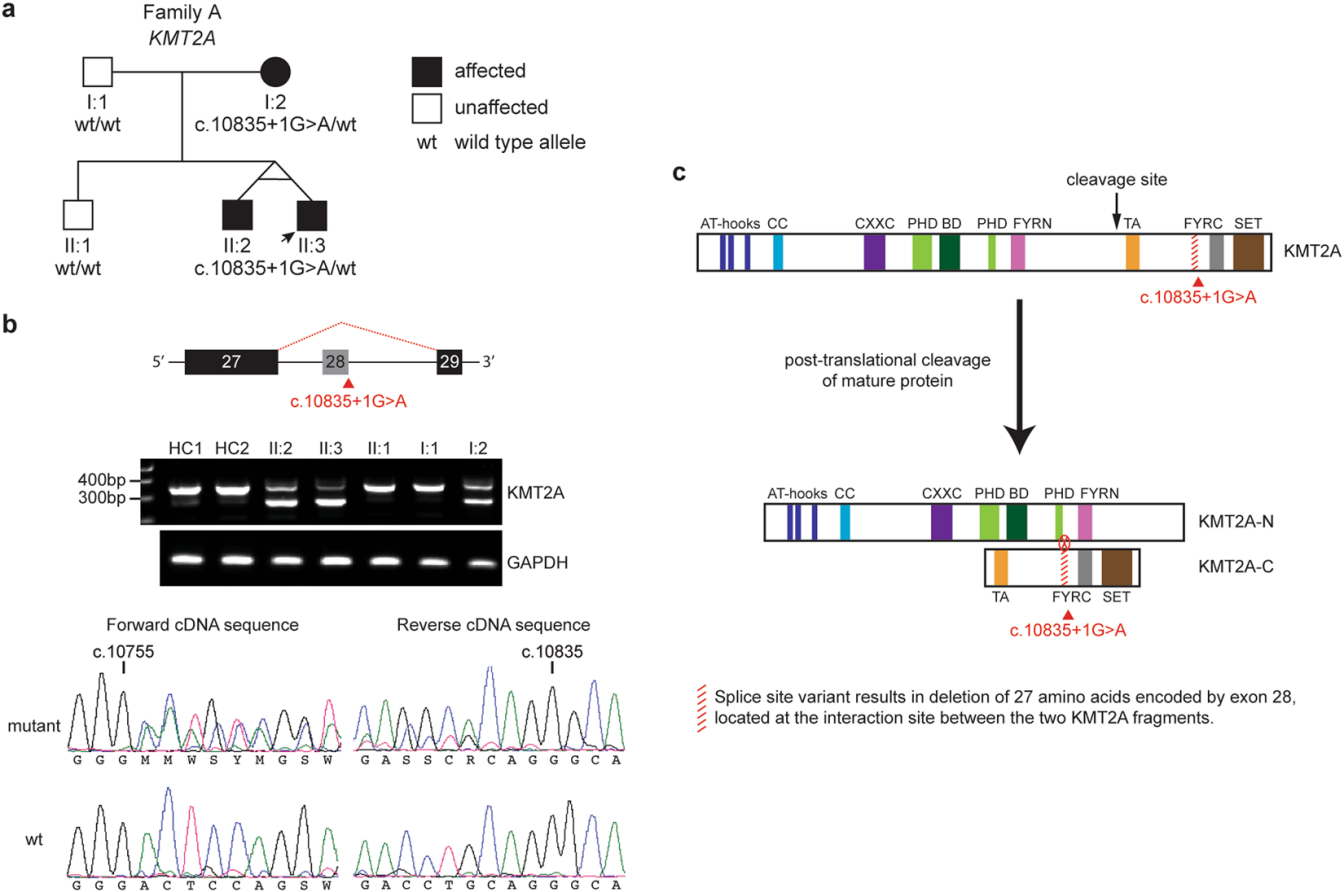
Family A with *KMT2A*-associated Wiedemann-Steiner syndrome (WSS). (**a**) Pedigree of family A. (**b**) Skipping of *KMT2A* exon 28. Gel electrophoresis of the *KMT2A* cDNA region containing exon 28 revealed a second shorter transcript in the three affected individuals. HC1 and HC2 represent two healthy controls; *GAPDH* was used as reference target. In-frame deletion of exon 28 was confirmed by cDNA sequencing; c.10755 and c.10835 indicate the start respectively stop position of exon 28. (**c**) KMT2A protein domains (adapted from ref. 9). KMT2A is cleaved in an N-terminal (KMT2A-N) and C-terminal (KMT2A-C) fragment, which form a non-covalently associated complex. Deletion of the amino acids encoded by exon 28 may disrupt the interaction site between the two fragments.

This study reports on two unrelated sibling pairs with recurrent respiratory tract infections and antibody deficiency categorized as CVID in early childhood. The family A monozygotic twin boys (Fig. 1a, II:2 and II:3) were born prematurely at 34 weeks gestational age to non-consanguineous, Belgian parents and are currently 11 years old. One of them (II:3) was born with bilateral inguinal hernia and hypospadias, which were attributed to his premature birth. From the first year of life, the twin boys suffered from recurrent upper and lower respiratory tract infections, often requiring antibiotics. At 2 months of age, patient II:3 developed severe pneumonia with respiratory arrest and heart failure. The latter led to the recognition of a patent *ductus arteriosus*, which was surgically ligated shortly thereafter. The postoperative course was complicated by severe respiratory distress requiring ventilation and systemic corticosteroids. Upon immunological evaluation, both patients II:2 and II:3 demonstrated panhypogammaglobulinemia, poor antibody responses to Pneumococcal polysaccharide vaccine, increased naive B cells, and very low memory B cells (Table 1). Additionally, both patients showed evidence of mild bronchiectasis on high-resolution computed tomography (HRCT) scan at 3.5 years of age.

**Table 1:** Routine immunological laboratory results of the family A patients with Wiedemann-Steiner syndrome.

	Patient II:2		Patient II:3		Patient I:2	
	Value	Reference range	Value	Reference range	Value	Reference range
**White blood cells**	*Age: 8 years*		*Age: 8 years*		*Age: 45 years*	
Total leukocytes (no./µL)	11930	6000–14000	7340	6000–14000	7210	3650–9300
Neutrophils (no./µL)	6980	2000–8000	3150	2000–8000	3450	1573–6100
Lymphocytes (no./µL)	3990	1500–7500	3480	1500–7500	2870	1133–3105
CD3+ T cells (no./µL)	2630	700–4200	2580	700–4200	2240	700–2100
CD3+ CD4+ T helper cells (no./µL)	1600	300–2000	1640	300–2000	1120	300–1400
CD45RA + naive CD4 + T cells (%)	82	46–77^†^	82	46–77^†^	44	NA
CD45RO+ memory CD4+ T cells (%)	12	13–30^†^	10	13–30^†^	50	NA
CD3+ CD8+ T cytotoxic cells (no./µL)	838	300–1800	800	300–1800	1060	200–1200
CD45RA+ naive CD8+ T cells (%)	80	63–92^†^	69	63–92^†^	26	NA
CD45RO+ memory CD8+ T cells (%)	12	4–21^†^	10	4–21^†^	73	NA
CD19+ B cells (no./µL)	798	200–1600	592	200–1600	287	100–500
IgD + CD27- naive B cells (%)	96	47.3–77.0^‡^	94	47.3–77.0^‡^	88	48.4–79.7^‡^
CD24 + + CD38++ transitional B cells (%)	19	4.6–8.3^‡^	10	4.6–8.3^‡^	14	0.9–5.7^‡^
IgD − CD27+ switched memory B cells (%)	1	10.9–30.4^‡^	1	10.9–30.4^‡^	6	8.3–27.8^‡^
IgD + CD27+ marginal zone B cells (%)	2	5.2–20.4^‡^	2	5.2–20.4^‡^	3	7.0–23.8^‡^
CD21^low^ CD38^low^ B cells (%)	2	2.3–10.0^‡^	3	2.3–10.0^‡^	2	1.6–10.0^‡^
CD3 − CD56 + CD16+ NK cells (no./µL)	479	90–900	244	90–900	344	90–600
Monocytes (no./µL)	690	700–1500	570	700–1500	780	247–757
Eosinophils (no./µL)	220	200–500	100	200–500	80	28–273
Basophils (no./µL)	30	10–100	20	10–100	10	6–50
Immunoglobulins*	*Age: 7 years*		*Age: 3 years*		*Age: 45 years*	
IgG (g/L)	3.6	4.70–10.5	2.8	4.70–9.30	7.6	7.0–16.0
IgG2 (g/L)	1.13	0.85–4.10	0.54	0.63–3.0	3.03	1.50–6.40
IgG3 (g/L)	0.265	0.13–1.42	0.176	0.13–1.26	0.307	0.20–1.10
IgM (g/L)	<0.2	0.27–0.63	<0.2	0.27–0.57	<0.18	0.40–2.48
IgA (g/L)	0.3	0.50–1.41	0.3	0.41–0.91	2.35	0.71–3.65
IgE (kU/L)	<4.4	0–90	<4.4	0–60	NA	
Specific antibody responses*	*Age: 7 years*		*Age: 3 years*		*Age: 45 years*	
*S*. *pneumoniae* polysaccharide IgG (Lab U)	NA		9	≥11: immune	NA	
*S*. *pneumoniae* polysaccharide IgG: specific IgG response to 3 serotypes (8, 9 N, 15B)	Insufficient antibody response	2x titer increase for at least 2 out of 3 serotypes	NA		Good antibody response	2x titer increase for at least 2 out of 3 serotypes
Tetanus IgG (IU/mL)	0.01	≥ 0.01: immune	0.03	≥ 0.01: immune	0.50	≥ 0.01: immune
Rubella IgG (IU/mL)	12	>10: immune	44	>10: immune	NA	
Measles IgG (mIU/mL)	350	>300: immune	1200	>300: immune	NA	
Mumps IgG (Lab U/mL)	270	>500: immune	540	>500: immune	NA	
Varicella Zoster IgG (mIU/mL)	620	>100: immune	1400	>100: immune	NA	
**Lymphocyte proliferation assay**	*Age: 3 years*		*Age: 3 years*			
Response to Concanavalin A	Normal	Compared to control	Normal	Compared to control	NA	
Response to Phytohemagglutinin	Normal	Compared to control	Normal	Compared to control	NA	
Response to Tetanus toxoid	Normal	Compared to control	Moderately reduced	Compared to control	NA	

The most recent, comprehensive and representative laboratory results are shown for each patient. Patients II:2 and II:3 were immunized according to the recommended Belgian childhood immunization schedule that, among others, included tetanus, measles, mumps, rubella and 7-valent conjugated pneumococcal vaccines. Patient I:2 had received a tetanus booster vaccine within the last 10 years. A polysaccharide (unconjugated) pneumococcal vaccine was given to patients II:2 and I:2 at time of immunological evaluation; patient I:2 had never received a pneumococcal vaccine before then. Patients II:2 and II:3 were not vaccinated against varicella zoster virus but had chickenpox in early childhood. NA: not available. *Measured when not receiving immunoglobulin replacement therapy. ^†^Reference values from Shearer *et al*.^27^. ^‡^Reference values from Piatosa *et al*.^28^.

**Figure 2:**
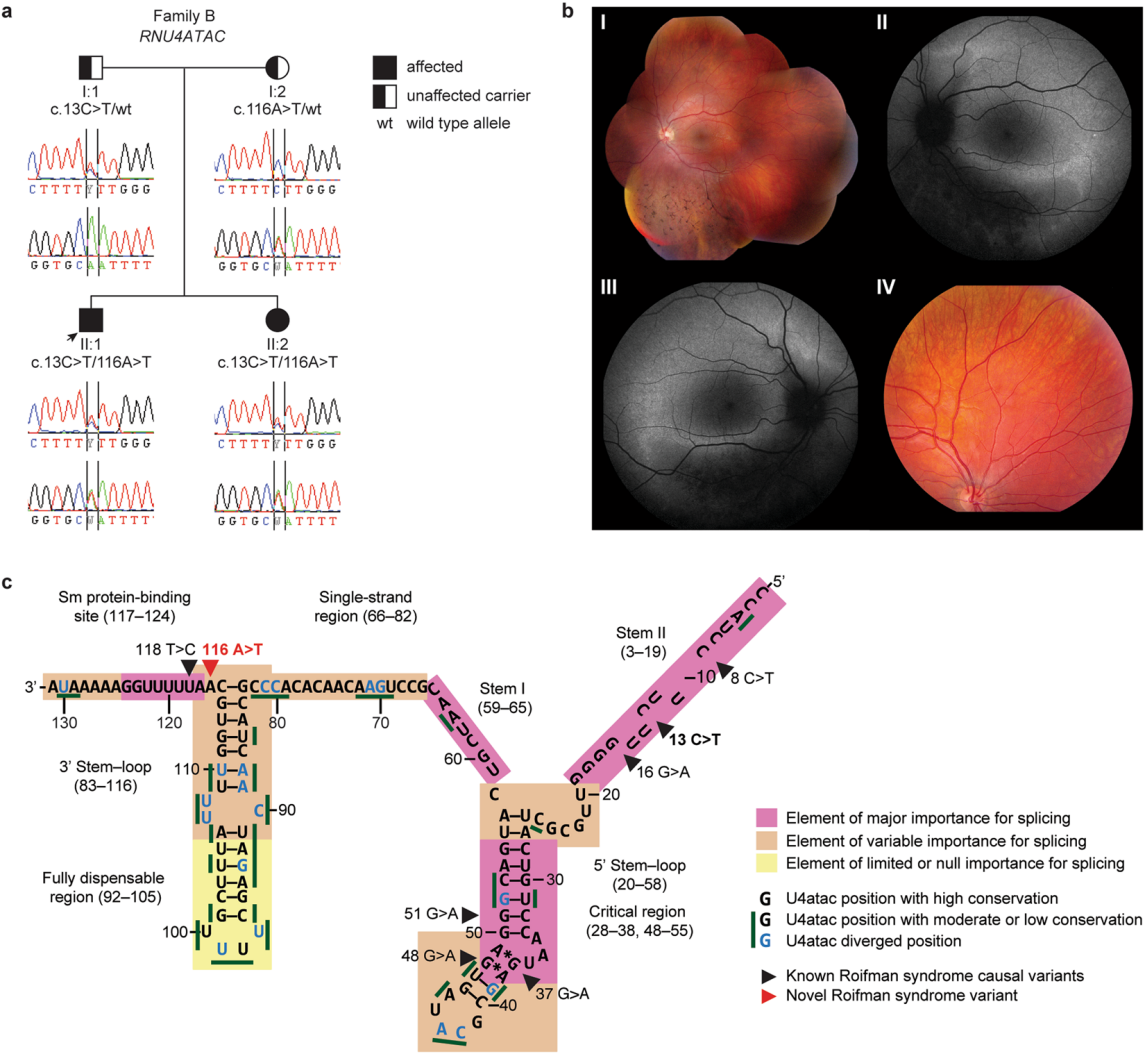
Family B with *RNU4ATAC*-associated Roifman syndrome (RS). (**a**) Pedigree of family B. (**b**) Representative retinal images of the RS patients. Panel I, composite retinal image of fundus of left eye (LE) of patient II:1: note inferior outer retinal atrophy with greyish hue and intraretinal pigment migration of the spicular type in inferior retina; mottled aspect of retinal pigment epithelium, more pronounced in inferotemporal area. Panel II, blue light autofluorescence image of LE of patient II:1 showing hyperautofluorescent delineation of inferior atrophic zone, as well as superior to optic disc, illustrating more widespread disease than can be seen on white light fundoscopic image only. Panel III, similar image of right eye (RE) of patient II:1 as in Panel II. Panel IV, fundus picture of detail of superonasal midperiphery of RE of patient II:2. Despite a normal full-field flash electroretinography, recent fundus examination at 14 years of age showed a mild mottling of pigment epithelium suggestive of early stage retinal dystrophy. (**c**) U4atac snRNA showing structural elements, conserved positions and location of variants associated with RS (adapted from ref. 8). The here-reported variant that has not been previously associated with RS is shown in red.

The family B brother and sister pair (Fig. 2a, II:1 and II:2) were born to healthy, non-consanguineous, Belgian parents and are currently 17 and 14 years of age respectively. They had recurrent upper and lower respiratory tract infections since the age of 18 months (II:1) and 3 years (II:2). The girl (II:2) also had recurrent gastroenteritis in early childhood and diffuse atopic eczema since infancy. Similar to the family A sibling pair, they had panhypogammaglobulinemia and poor global antibody responses to Pneumococcal polysaccharide vaccine (Table 2). Serotype-specific Pneumococcal antibody responses were not evaluated. In contrast to the family A twins, the family B siblings demonstrated severe B cell lymphopenia with normal switched memory B cell percentages and increased CD21^low^ B cell levels (Table 2). HRCT scan in patient II:1 at the age of 7.5 years displayed marked bronchiectasis, whereas his sister (II:2) only showed discrete bronchiectasis at a similar age.

**Table 2:** Routine immunological laboratory results of the family B patients with Roifman syndrome.

	Patient II:1		Patient II:2	
	Value	Reference range	Value	Reference range
**White blood cells**	*Age: 14 years*		*Age: 11 years*	
Total leukocyte count (no./µL)	7720	4500–12000	9800	4500–12000
Neutrophils (no./µL)	5290	2500–8000	6080	2500–8000
Lymphocytes (no./µL)	1190	1500–6500	2410	1500–6500
CD3+ T cells (no./µL)	940	800–3500	1740	800–3500
CD3 + CD4+ T helper cells (no./µL)	643	400–2100	1080	400–2100
CD45RA+ naive CD4+ T cells (%)	55	33–66^†^	68	33–66^†^
CD45RO+ memory CD4+ T cells (%)	38	18–38^†^	27	18–38^†^
CD3 + CD8+ T cytotoxic cells (no./µL)	274	200–1200	603	200–1200
CD45RA+ naive CD8+ T cells (%)	60	61–91^†^	67	61–91^†^
CD45RO+ memory CD8+ T cells (%)	33	4–23^†^	22	4–23^†^
CD19+ B cells (no./µL)	36	200–600	48	200–600
IgD + CD27- naive B cells (%)	77	51.3–82.5^‡^	70	51.3–82.5^‡^
CD24 + + CD38++ transitional B cells (%)	15	1.4–13.0^‡^	5	1.4–13.0^‡^
IgD-CD27+ switched memory B cells (%)	9	8.7–25.6^‡^	10	8.7–25.6^‡^
IgD + CD27+ marginal zone B cells (%)	4	4.6–18.2^‡^	1	4.6–18.2^‡^
CD21^low^ CD38^low^ B cells (%)	22	2.7–8.7^‡^	21	2.7–8.7^‡^
CD3-CD56 + CD16+ NK cells (no./µL)	179	70–1200	554	70–1200
Monocytes (no./µL)	910	500–1000	960	500–1000
Eosinophils (no./µL)	290	100–500	230	100–500
Basophils (no./µL)	20	10–100	90	10–100
Immunoglobulins*	*Age: 3 years*		*Age: 6 years*	
IgG (g/L)	3.8	4.7–9.3	4.4	4.7–10.5
IgG2 (g/L)	0.53	0.63–3.0	0.49	0.85–4.1
IgG3 (g/L)	0.021	0.13–1.26	0.242	0.13–1.42
IgM (g/L)	<0.2	0.27–0.57	0.3	0.27–0.63
IgA (g/L)	0.3	0.41–0.91	<0.3	0.5–1.41
IgE (kU/L)	<4.4	0–60	<4.4	0–90
Isohemagglutinins*	*Age: 3 years*			
ABO blood type	O		NA	
Anti-A IgM	Negative	Positive	NA	
Anti-B IgM	Negative	Positive	NA	
Specific antibody responses*	*Age: 3 years*		*Age: 6 years*	
*S*. *pneumoniae* polysaccharide IgG (Lab U)	<3	≥ 11: immune	7	≥ 11: immune
Tetanus IgG (IU/mL)	0.03	≥ 0.01: immune	1	≥ 0.01: immune
Rubella IgG (IU/mL)	<8	>10: immune	NA	
Measles IgG (mIU/mL)	<150	>300: immune	NA	
Mumps IgG (Lab U/mL)	<230	>500: immune	NA	
Varicella zoster IgG (mIU/mL)	360	>100: immune	1400	>100: immune
**Lymphocyte proliferation assay**	*Age: 9 years*		*Age: 6 years*	
Response to Concanavalin A	Normal	Compared to control	Normal	Compared to control
Response to Phytohemagglutinin	Normal	Compared to control	Normal	Compared to control
Response to Tetanus toxoid	Normal	Compared to control	Normal	Compared to control

The most recent, comprehensive and representative laboratory results are shown for each patient. Both patients were immunized according to the recommended Belgian childhood immunization schedule that, among others, included tetanus, measles, mumps, rubella and 7-valent conjugated pneumococcal vaccines. A polysaccharide (unconjugated) pneumococcal vaccine was given at time of immunological evaluation. The patients were not vaccinated against varicella zoster virus but had chickenpox in early childhood. NA: not available. *Measured when not receiving immunoglobulin replacement therapy. ^†^Reference values from Shearer *et al*.^27^. ^‡^Reference values from Piatosa *et al*.^28^.

In both the family A and B sibling pairs, the clinical presentation and laboratory findings in the first years of life were reminiscent of a CVID phenotype. All patients are currently doing well under regular Ig replacement therapy, antiflogistic maintenance treatment with azithromycin and intermittent inhaled corticosteroids and/or short-acting beta-agonists therapy.

### Extra-immunological features raised suspicion of syndromic PID

Although both sibling pairs first presented with a phenotype resembling CVID, with time they gradually demonstrated additional clinical features not typically associated with CVID (Tables 3 and 4). The family A twin boys developed a third degree atrioventricular block for which a pacemaker was implanted at 5 (II:3) and 6.5 (II:2) years of age respectively. In the following years, the boys showed increasing evidence of mild intellectual disability. In retrospect, patient II:3 had signs of mild developmental delay during the first years of life. Both twins also demonstrated poor weight gain and growth retardation, albeit to a limited extent. Around the age of 9 years, dysmorphic facial features became more conspicuous (Table 3). Interestingly, the twins’ mother (I:2) had congenital urogenital tract anomalies consisting of a unicornuate uterus and a unique left ovary, fallopian tube and kidney. Moreover, since childhood, she had suffered from right unilateral sensorineural hearing loss as well as recurrent sinusitis and bronchitis frequently requiring antibiotics. At that time, genetic or immunological testing had never been performed in the mother as she deemed herself to be in good general health. The twins’ older brother (II:1) and father (I:1) had an uneventful medical history.

**Table 3:** Comparison of the family A patients with published cases of *KMT2A*-associated Wiedemann-Steiner syndrome.

Clinical features^†^	18 published patients^7, 9–14^	Present study		
		Patient II:2	Patient II:3	Patient I:2
Gender	8 M, 10 F	M	M	F
Age at last examination (years)	1–24	11	11	46
Short stature	18/18	+	+	+
*Craniofacial features*				
Microcephaly	2/7	−	−	−
Mild macrocephaly	NA	+	+	−
Hypertelorism, telecantus	9/17	+	+	+
Down-slanted palpebral fissures	14/16	+	+	+
Vertically narrow palpebral fissures	13/17	+	+	+
Strabismus	4/17	−	−	−
Thick eyebrows	14/17	+	+	+
Wide nasal bridge	16/18	+	+	+
Broad nasal tip	11/17	+	+	+
Long philtrum	2/12	−	−	−
Thin upper lip	6/12	+	+	+
Low-set ears	2/12	+	+	+
Abnormal dentition, hypodontia	5/9	−	−	−
High palate	4/8	+	+	+
Micrognathia	7/11	+	+	+
*Musculoskeletal features*				
Advanced bone age	1/16	NA	NA	NA
Small hands and feet	5/17	+	+	+
Fleshy hands and feet	3/7	+	+	+
Clinodactyly	8/18	−	+	+
Congenital hip dysplasia	2/17	−	−	−
Muscular hypotonia	9/18	−	−	−
*Dermatological features*				
Thick hair	14/17	+	+	+
*Hypertrichosis cubiti*	13/18	−	−	−
Hypertrichosis back and/or lower limbs	16/18	−	−	−
*Neurological features*				
Developmental or psychomotor delay	18/18	−	+	NA
Intellectual disability	16/17	+	+	+
Autism	2/12	−	−	−
Aggressive behavior	4/13	−	−	−
Hyperactivity	2/12	−	−	−
Seizures	1/7	−	−	−
*Internal organ anomalies*				
Cardiovascular anomalies	3/17	+	+	−
Urogenital anomalies	4/17	−	+	+
Intestinal anomalies	4/11	−	−	−
Feeding difficulties	10/18	+	+	−
*Immunodeficiency features*				
Antibody deficiency	1/1	+	+	+
Respiratory tract infections	2/17	+	+	+
Urinary tract infections	4/18	−	−	−
Bronchiectasis	NA	+	+	NA

NA: not available. ^†^Not all clinical features have been ascertained in all previously published patients. Adapted from Stellacci *et al*.^7^.

**Table 4:** Comparison of the family B patients with published cases of *RNU4ATAC*-associated Roifman syndrome.

Clinical features	6 published patients^8^	Present study	
		Patient II:1	Patient II:2
Gender	5 M, 1 F	M	F
Age at last examination (years)	NA	17	14
*Growth retardation*			
Prenatal, intra-uterine growth retardation	6/6	NA	NA
Postnatal growth retardation	6/6	+	+
*Craniofacial features*			
Mild microcephaly	5/6	+	+
Long philtrum	6/6	+	+
Thin upper lips	6/6	+	+
Narrow, tubular and upturned nose	6/6	+	+
*Ophthalmological features*			
Retinal dystrophy	3/6	+	+
*Musculoskeletal features*			
Epiphyseal dysplasia	6/6	+	−
Vertebral changes	3/6	+	−
Coxa vara	NA	+	−
Agenesis of anterior cruciate ligaments	NA	+	−
Agenesis of 12^th^ ribs	NA	+	−
Short metacarpals	6/6	+	−
5^th^ digit clinodactyly	4/6	−	−
Brachydactyly	6/6	+	−
Transverse palmar crease	5/6	−	−
Muscular hypotonia	5/6	−	−
*Neurological features*			
Intellectual disability, cognitive delay	5/6	−	−
Sensorineural hearing loss	1/6	−	−
*Internal organ anomalies*			
Noncompaction of the myocardium	1/6	−	−
Ventricular septum defect (VSD)	1/6	−	−
Lung hypoplasia	NA	−	+
*Immunodeficiency and atopic features*			
Antibody deficiency	6/6	+	+
Hepatosplenomegaly	5/6	−	−
Bronchiectasis	NA	+	+
Eczema	3/6	−	+

NA: not available.

Analogously, the boy in family B (II:1) initially displayed subtle syndromic features, such as mild growth retardation, that appeared more pronounced over time. At the age of seven years, diverse skeletal abnormalities including spondyloepiphyseal dysplasia were detected (Table 4). At the same age, he was also found to have slowly progressive retinal dystrophy (Fig. 2b, Panels I-III) with decreased rod function but near-normal cone function on full-field flash electroretinography. Antibody deficiency in combination with skeletal and ophthalmological features led to the clinical suspicion of RS in the boy. However, his sister (II:2) had no radiographic sings of spondyloepiphyseal or hip dysplasia nor retinal dystrophy. Moreover, RS was originally presumed to be an X-linked recessive condition, although no causal gene had been identified^16^. When the family B girl (II:2) was about 9 years old, Gray *et al*. reported the first female patient with RS, having a skewed X-inactivation and a milder phenotype than her affected brother^17^. Subsequently, we hypothesized that patient II:2 might be a manifesting heterozygote of RS, which could be compatible with her milder extra-immunological phenotype at that time.

### Cytogenetic and cytogenomic analyses were negative in both sibling pairs

In the family A twins, conventional G-banding karyotype, fluorescent *in situ* hybridization for region 22q11.2 and subtelomeric screening were normal. Furthermore, microarray-based comparative genomic hybridisation analysis in both sibling pairs did not demonstrate copy number variations.

### Whole exome sequencing (WES) uncovers WSS in family A

Since no specific genetic syndrome was suspected in family A, WES was performed in patient II:2 and both parents. This revealed a heterozygous splice site variant in *KMT2A* (NM_001197104:c.10835 + 1 G > A), present in the twins (II:2, II:3) as well as in the mother (I:2) (Fig. 1a). The variant is not reported in public or in-house databases. The *KMT2A* nucleotide substitution is situated in the splice donor site of intron 28. *In silico* splicing prediction tools suggested complete loss of the splice donor site resulting in exon 28 skipping and an in-frame deletion of 81 bp, which was confirmed by analyses on cDNA derived from patients’ PBMCs (Fig. 1b). Mature KMT2A protein is physiologically cleaved in an N-terminal (KMT2A-N) and C-terminal (KMT2A-C) fragment, which together form a non-covalently associated complex (Fig. 1c)^15, 18^. Complex formation is necessary for stability and subnuclear localization of the protein^18^. The amino acids encoded by exon 28 are part of the interaction site between KMT2A-N and KMT2A-C (Fig. 1c)^15^. It has been shown that disrupting the interaction between the two fragments causes degradation of the KMT2A-N fragment and loss of protein function^18^. The KMT2A-N fragment was only very weakly detectable by western blot on PBMC lysates, however, and could therefore not be reliably interpreted (data not shown). Subsequent investigations in the mother demonstrated mild intellectual disability, undetectable serum IgM, and reduced switched memory B cells (Table 1). Taken together, the c.10835 + 1 G > A variant in *KMT2A* indicates a molecular diagnosis of WSS in the twin brothers and their mother.

### Targeted sequencing confirms the diagnosis of RS in family B

In family B, WES was unable to identify a potentially disease-causing variant. In 2015, biallelic mutations in *RNU4ATAC* were identified in patients with RS^8^. Since *RNU4ATAC* is a noncoding snRNA gene, possible variants would have been missed with WES. Indeed, subsequent Sanger sequencing of *RNU4ATAC* revealed compound heterozygous variants in both siblings (c.13 C > T and c.116 A > T) that segregated in the parents (Fig. 2a). The c.13 C > T variant (rs559979281) had been previously reported in RS (Fig. 2c)^8^. The c.116 A > T variant has, to our knowledge, not yet been associated with human disease. The public database gnomAD (Genome Aggregation Database) contains two heterozygotes for the c.116 A > T variant (allele frequency of 0.00001531) but no homozygotes. Importantly, the c.116 A > T variant is located in a highly conserved position involved in splicing activity (Fig. 2c)^8^. Furthermore, position 116 is immediately adjacent to the Sm protein-binding site, which is a highly conserved structural element essential in splicing activity and previously implicated in RS (Fig. 2c)^8^. Together, the *RNU4ATAC* genotype confirms the diagnosis of RS in the family B siblings.

### Immunological abnormalities in the WSS and RS patients

**Figure 3:**
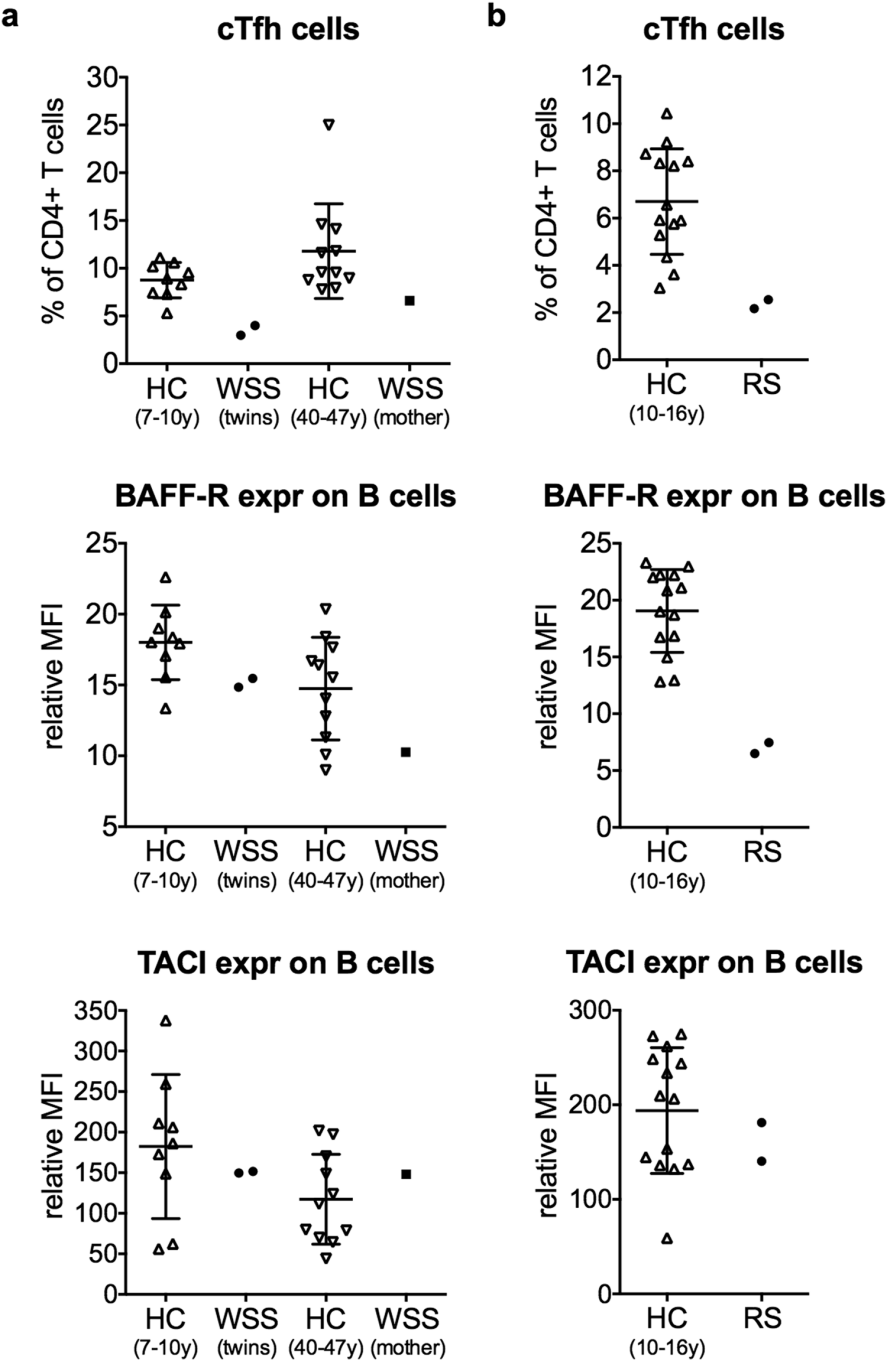
cTfh cells, BAFF-R expression and TACI expression in WSS and RS patients. (**a**) Family A patients with *KMT2A*-associated Wiedemann-Steiner syndrome (WSS). The twins (II:2, II:3) were 8 years old and the mother (I:2) was 43 years old at time of analysis. (**b**) Family B patients with *RNU4ATAC*-associated Roifman syndrome (RS). At time of analysis, the patients (II:1, II:2) were 14 and 11 years old, respectively. Flow cytometric immunophenotyping was performed on patients’ PBMCs in comparison with age-matched healthy controls (HC). T cells were gated as CD3^+^ and B cells as CD19^+^CD20^+^ in total PBMCs. Circulating follicular helper T (cTfh) cells were gated as CXCR5^+^CD45RO^+^ in CD4^+^ T cells. BAFF-R and TACI expression were measured on B cells. Relative mean fluorescence intensity (MFI) was calculated by dividing the MFI of the positive population by the MFI of the Fluorescence Minus One (FMO) population. Graphs of the HC groups represent mean ± standard deviation. BAFF-R: B cell activating factor-receptor, cTfh: circulating follicular helper T, expr: expression, TACI: transmembrane activator and calcium modulator and cyclophilin ligand interactor.

Because of the prominent immunodeficiency in both sibling pairs, we performed flow cytometric analysis of B and T lymphocyte subsets as previously described^19^. Interestingly, all patients from families A and B had decreased circulating follicular helper T (cTfh) cells (Fig. 3a,b). Tfh cells play an essential role in the formation of antibody-producing plasma cells and memory B cells^20^. Furthermore, the two RS patients showed markedly reduced expression levels of B cell activating factor-receptor (BAFF-R), a receptor important in peripheral B cell survival (Fig. 3b)^21^. The WSS patients had normal BAFF-R levels (Fig. 3a). Expression of transmembrane activator and calcium modulator and cyclophilin ligand interactor (TACI), a receptor related to BAFF-R, was normal in both the WSS and RS patients (Fig. 3a,b)^21^. For all patients, the alterations in naive and memory lymphocyte subsets (Supplementary Figs 1–4) corresponded with those seen in the routine laboratory assessment (Table 1). Other examined B and T cell populations fell within the range of the age-matched healthy controls (Supplementary Figs 1–4).

## Discussion

We report two sibling pairs with an early-onset CVID phenotype as primary and cardinal presentation of WSS and RS: recurrent sinopulmonary infections, panhypogammaglobulinemia, reduced polysaccharide vaccine responses, and aberrant peripheral B cell subsets^1^. Because extra-immunological features were initially subtle and only became conspicuous with age, the establishment of an accurate diagnosis was significantly delayed. Therefore, we recommend to proactively evaluate all paediatric patients with a CVID phenotype for extra-immunological syndromic features, especially when presenting at an early age. In particular, diagnostic workup should include evaluation by a clinical geneticist, in addition to orthopedic, cardiologic, neurologic, urogenital and ophthalmologic assessment. To reach a conclusive diagnosis, genetic testing should be performed, varying from targeted testing of a specific disease gene to WES. Here, the diagnosis of WSS in family A was only confirmed upon WES in affected family members^7, 9^. In family B on the other hand, WES failed to reveal the causal genetic defect because this was located in a noncoding gene^8^. Targeted testing of the known disease gene for RS allowed to identify the underlying mutations and to provide a definite diagnosis^8^. Of note, if WES does not identify a genetic defect and there is no known disease gene, whole genome sequencing should be undertaken^22^.

With this study, we extend the phenotypical and mutational spectrum of both *KMT2A*-associated WSS and *RNU4ATAC*-associated RS^7–14^. In family A, we identified a novel heterozygous splice site mutation in *KMT2A* causing in-frame deletion of exon 28. This deletion likely disrupts the stabilizing interaction site between the N- and C-terminal KMT2A fragments, resulting in loss of protein function^18^. So far, we were unable to confirm this on a protein level because the KMT2A-N protein fragment was not reliably detectable by western blot. Further studies on protein level are planned in the future. Family A is the first published kindred to show autosomal dominant transmission of *KMT2A*-associated WSS in multiple generations, as previously reported cases were either sporadic or parents were unavailable^7^. Remarkably, the characteristic hypertrichosis of elbows, back and/or lower limbs was absent in the here-reported WSS patients, confirming previous literature that this feature may not be as pathognomonic as initially believed^7, 9, 11, 12^. Interestingly, *KMT2A*-associated WSS shows phenotypical overlap with Kabuki syndrome type 1 caused by heterozygous mutations in the related gene *KMT2D* ^23^. Over 80% of patients with *KMT2D*-associated Kabuki syndrome develop defects in terminal B cell differentiation resulting in antibody deficiency^23^. Similarly, the here-described WSS patients demonstrated a block in terminal B cell differentiation evidenced by a relative increase in transitional and naive B cells and a relative decrease in switched memory B cells. Moreover, they had reduced levels of cTfh cells, which play a pivotal role in terminal B cell differentiation^20^. Decreased cTfh cells have, to our knowledge, not been previously reported in WSS or Kabuki syndrome. It would be interesting to investigate cTfh cells in additional patients with *KMT2A*-associated WSS and *KMT2D*-associated Kabuki syndrome as this may help elucidate the underlying pathophysiology of the antibody deficiency. In summary, humoral immune deficiency in patients with WSS reported by us and by Stellacci *et al*.^7^, and antibody deficiency in patients with heterozygous mutations in the related *KMT2D* gene^23^, strongly suggest a previously unknown role for *KMT2A* in B cell biology that may be related with T helper cell function.

In family B, we identified rare compound heterozygous mutations in the noncoding *RNU4ATAC* gene, of which one mutation (c.116 A > T) had not been associated with RS before^8^. Although it was not initially apparent, with time the boy showed typical features of RS including spondyloepiphyseal dysplasia and retinal dystrophy^8^. Curiously, he also demonstrated bilateral agenesis of the anterior cruciate ligaments and the 12^th^ ribs, which are not typically seen in RS^8^. Spondyloepiphyseal or hip dysplasia have not yet been documented in the girl, currently 14 years old, although she displays mild growth retardation. Only recently, she was found to have mild fundus abnormalities suggestive of early stage retinal dystrophy. Note that retinal dystrophy was already evident in her brother at 7 years of age. It would be interesting to investigate why the girl has a milder phenotype than her brother. Since U4atac snRNA plays a role in minor intron splicing, it would be valuable to conduct RNA sequencing analysis in the two siblings and check for possible differences in intron retention^8^. Detailed immunological workup in the RS patients revealed markedly decreased BAFF-R expression on B cells. To our knowledge, this finding has not been previously published. As BAFF-R signalling is important for survival of B cells in the peripheral blood, this may provide an important clue towards the B cell lymphopenia seen in RS patients^21^. Moreover, analogous to the WSS patients, the RS siblings demonstrated decreased levels of cTfh cells, which may further compromise B cell differentiation and antibody production^20^.

In conclusion, we here illustrate that a CVID phenotype can be the initial presentation of WSS and RS in early childhood while hallmark extra-immunological characteristics may be less prominent. With this, we highlight the importance of pursuing a genetic diagnosis in paediatric patients with an early-onset CVID phenotype, as this has important implications in terms of counselling, follow-up and screening for complications associated with the specific disorder.

## Methods

### Statement

All experiments and methods were carried out in accordance with relevant guidelines and regulations. The research protocol and all experimental protocols were approved by the ethical committee of Ghent University Hospital (2012/593). All reported subjects provided written informed consent for participation in the study, in accordance with the 1975 Helsinki Declaration.

### Cytogenetic analyses

Microarray-based comparative genomic hybridization (array CGH) was performed on the affected sibling pairs of families A and B using the SurePrint G3 Human CGH Microarray Kit according to manufacturer’s instructions (Agilent Technologies). Results were analyzed with arrayCGHbase^24^. Karyotype analysis was performed on the family A twins using the conventional G-banding technique. To screen the family A twins for submicroscopic subtelomeric rearrangements, multiplex ligation-dependent probe amplification (MLPA) analysis was performed using SALSA P070 and SALSA P036C probe mixes according to manufacturer’s instructions (MRC-Holland). To examine the family A twins for 22q11.2 deletion, fluorescence *in situ* hybridization (FISH) analysis was performed using the DiGeorge Region Probe Set – LSI TUPLE 1 SpectrumOrange/LSI ARSA SpectrumGreen according to manufacturer’s instructions (Vyvis).

### WES

Genomic DNA was isolated from whole blood leukocytes using the Puregene DNA isolation kit (Qiagen) according to manufacturer’s instructions. Whole exome enrichment was performed with the SureSelectXT Human All Exon V5 + UTRs kit (Agilent Technologies). Paired-end massively parallel sequencing (100 cycles) was performed on a NextSeq 500 (Illumina). Read mapping against the human genome reference sequence (NCBI, GRCh37), and post-mapping duplicate read removal, quality-based variant calling and coverage analysis were performed with CLC Genomics Workbench v6.0.4 (CLC bio). Sequencing coverage is summarized in Supplementary Table S1. Called variants with coverage ≥3 were annotated with Alamut Batch (Interactive Biosoftware). Only variants with population frequencies less than 10% were considered, according to public databases NCBI dbSNP (http://www.ncbi.nlm.nih.gov/projects/SNP/), NHLBI Exome Sequencing Project (http://evs.gs.washington.edu/EVS/), ExAC Browser (http://exac.broadinstitute.org/), and 1000 Genomes Project Browser (http://browser.1000genomes.org/). Variants were further prioritized based on allele frequency, functional prediction scores, nucleotide conservation scores and biological relevance^25^. Both Mendelian and non-Mendelian inheritance patterns were taken into account. Afterwards, variants of interest were evaluated using Alamut Visual mutation interpretation software v2.7 rev. 1 (Interactive Biosoftware), Ingenuity Variant Analysis (QIAGEN, 2015 Release Spring), CADD scores v1.3 (http://cadd.gs.washington.edu/home), genome Aggregation Database (gnomAD) Browser (http://gnomad.broadinstitute.org), literature search, segregation analysis in available family members, and frequency in an in-house database containing variants of more than 1000 exomes at time of analysis.

### Sanger sequencing of genomic DNA

DNA templates (GRCh37/hg19) of *KMT2A* (NM_001197104) and *RNU4ATAC* (NR_023343) were obtained from UCSC Genome Browser (https://genome.ucsc.edu). Primers for amplification and sequencing were designed with Primer3Plus^26^. For *KMT2A* exon 28 and adjacent intron-exon borders (family A): forward primer 5′-CAACCCACAAGGGTGTCTTC-3′ and reverse primer 5′-GCCCGGCTAATTCTTTTTGT-3′. For the unique exon and intron-exon borders of *RNU4ATAC* (family B): forward primer 5′-TGGAGGCTGGAGGTAAGCTA-3′ and reverse primer 5′-TGAGGTGCAAAGACCTACTGAA-3′. Genomic DNA was amplified by PCR using the specific primers and KAPA2G Robust Hotstart Ready Mix (KAPA Biosystems). PCR products were enzymatically purified with Exonuclease I and Antartic phosphatase (both New England BioLabs Inc.). Purified PCR products were sequenced using the BigDye Terminator v3.1 Cycle Sequencing kit (Applied Biosystems) on a 3730xl DNA Analyzer (Applied Biosystems). Sequence reads were analyzed with SeqScape v2.5 (ThermoFisher Scientific).

### RNA extraction, cDNA synthesis and confirmation of skipping of exon 28 in *KMT2A*

Total RNA was isolated from total PBMCs of all family A members and two control subjects by use of the RNeasy Plus Mini Kit (Qiagen) and reverse transcribed using the iScript cDNA synthesis kit (Bio-Rad), according to manufacturer’s instructions. The cDNA template (GRCh37/hg19) of *KMT2A* (NM_001197104) was obtained from UCSC Genome Browser (https://genome.ucsc.edu). Primers for amplification and sequencing of exon 28 and adjacent coding regions were designed with Primer3Plus^26^: forward primer 5′-AACCCAAACCAAAAACCAAAC-3′ and reverse primer 5′-CATCAGTGGGGAGCTGAAAT-3′. *GAPDH* was used as a reference target: forward primer 5′-CAGCCTCAAGATCATCAGCA-3′ and reverse primer 5′-TGTGGTCATGAGTCCTTCCA-3′. PCR amplification was performed by use of GoTaq Hot Start Colorless Master Mix (Promega). PCR products were analyzed on a 2% agarose gel in 1x TBE buffer (Quality Biological Inc). SYBR Safe (Invitrogen) signals were captured with a Gel Doc EZ Imager system (Bio-Rad). In addition, purified PCR products were Sanger sequenced using the BigDye Terminator v3.1 Cycle Sequencing kit (Applied Biosystems) on a 3130xL Genetic Analyzer (Applied Biosystems). Sequence reads were analyzed with SeqMan (DNAStar).

### Flow cytometric analysis of PBMCs

Immunophenotyping was performed on PBMCs of patients and age-matched healthy controls. PBMCs were isolated from EDTA whole blood by Ficoll-Paque density gradient centrifugation and cryopreserved at −150 °C. Thawed PBMCs were stained with fixable viability dye 506 (eBioscience) and fluorescently labeled monoclonal antibodies under saturation conditions as previously described^19^. Cells were acquired on an LSR Fortessa flow cytometer (BD Biosciences). Data were analyzed with FlowJo version X (Tree Star Inc.).

## Electronic supplementary material

Supplementary information
