# Supplementary material for: Early-onset primary antibody deficiency resembling common variable immunodeficiency challenges the diagnosis of Wiedeman-Steiner and Roifman syndromes
Source: Sci Rep. 2017 Jun 16;7:3702. doi: 10.1038/s41598-017-02434-4 (PMC5473876; doi:10.1038/s41598-017-02434-4)
Supplement: Supplementary file 1 — Supplementary information [file 41598_2017_2434_MOESM1_ESM.pdf]

SUPPLEMENTARY INFORMATION

Early-onset primary antibody deficiency resembling common variable immunodeficiency challenges the diagnosis of Wiedeman-Steiner and Roifman syndromes

Delfien J. Bogaert, MD[1,2,3,4], Melissa Dullaers, PhD[1,4,5], Hye Sun Kuehn, PhD[6], Bart P. Leroy, MD, PhD[3,7,8], Julie E. Niemela, MSc[6], Hans De Wilde, MD[9], Sarah De Schryver, MD[10], Marieke De Bruyne, MSc[3], Frauke Coppieters, PhD[3], Bart N. Lambrecht, MD, PhD[4,5,11], Frans De Baets, MD, PhD[2], Sergio D. Rosenzweig, MD, PhD[6], Elfride De Baere, MD, PhD[3,†], Filomeen Haerynck, MD, PhD[1,2,†,*]

Content

Supplementary Table 1. WES coverage depth.
Supplementary Figure 1. Flow cytometric phenotyping of the family A patients: B cell subsets.
Supplementary Figure 2. Flow cytometric phenotyping of the family A patients: T cell subsets.
Supplementary Figure 3. Flow cytometric phenotyping of the family B patients: B cell subsets.
Supplementary Figure 4. Flow cytometric phenotyping of the family B patients: T cell subsets.

Supplementary Table 1. WES coverage depth.

Family	Patient	Coverage depth				
		Average coverage	% of regions ≥ 5 x	% of regions ≥ 10 x	% of regions ≥ 20 x	% of regions ≥ 40 x
A	I:1	74.8 x	98.79%	97.75%	93.52%	75.81%
A	I:2	67.9 x	98.59%	97.39%	92.48%	71.62%
A	II:3	67.6 x	98.69%	97.47%	92.42%	71.62%
B	I:1	61.5 x	96.12%	93.80%	86.31%	63.60%
B	I:2	73.7 x	95.82%	93.82%	88.28%	71.08%
B	II:1	76.7 x	98.90%	97.85%	93.28%	75.34%
B	II:2	65.9 x	95.94%	93.75%	87.09%	67.07%

Coverage depth is calculated for all regions enriched by the SureSelectXT Human All Exon V5+UTRs kit (Agilent Technologies, CA, USA), using CLC Genomics Workbench v6.0.4 (CLC bio, MA, USA).

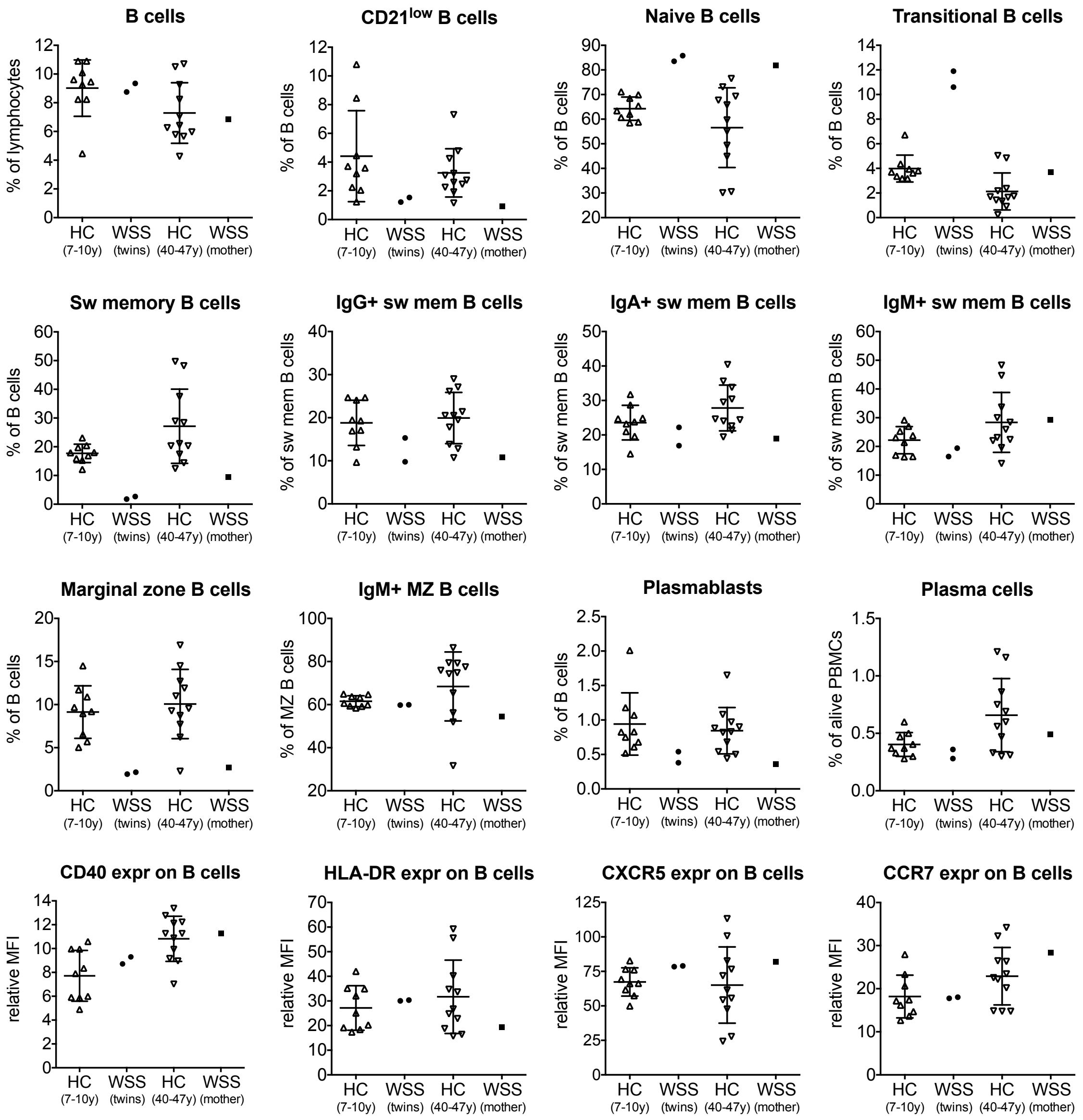

Supplementary Figure 1. Flow cytometric phenotyping of the family A patients: B cell subsets.
Flow cytometric immunophenotyping was performed on PBMCs of the 3 patients with *KMT2A*-associated Wiedemann-Steiner syndrome (WSS) in comparison with age-matched healthy controls (HC). At time of analysis, the twins and the mother were 8 and 43 years old, respectively. For details on gating, see reference 19. Relative mean fluorescence intensity (rMFI) was calculated by dividing the MFI of the positive population by the MFI of the Fluorescence Minus One (FMO) population. Graphs of HC group represent mean ± standard deviation. MZ, marginal zone; sw (mem), switched (memory); expr, expression.

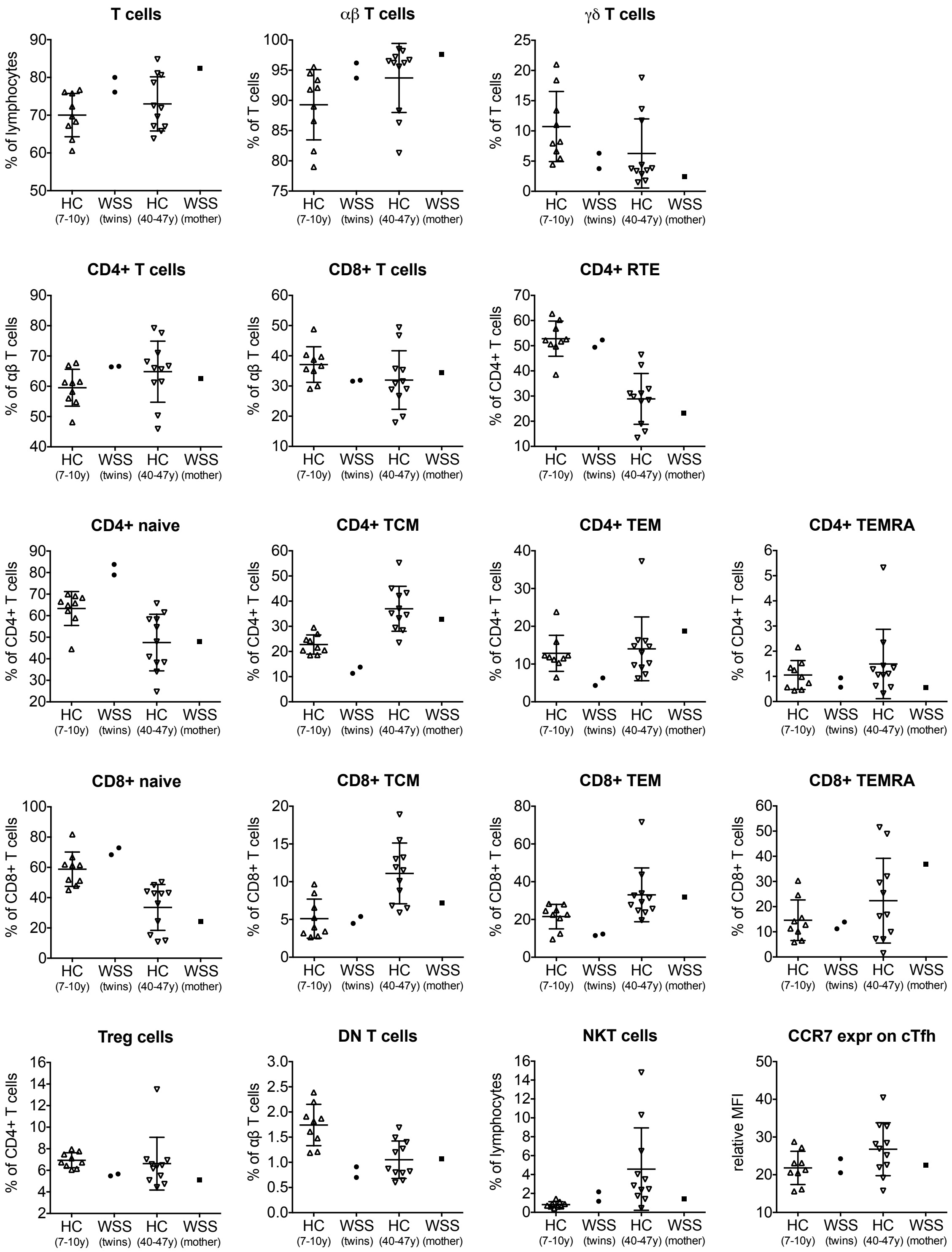

Supplementary Figure 2. Flow cytometric phenotyping of the family A patients: T cell subsets.
Flow cytometric immunophenotyping was performed on PBMCs of the 3 patients with KMT2A-associated Wiedemann-Steiner syndrome (WSS) in comparison with age-matched healthy controls (HC). At time of analysis, the twins and the mother were 8 and 43 years old, respectively. For details on gating, see reference 19. Relative mean fluorescence intensity (rMFI) was calculated by dividing the MFI of the positive population by the MFI of the Fluorescence Minus One (FMO) population. Graphs of HC group represent mean ± standard deviation. RTE, recent thymic emigrants; TCM, central memory T; TEM, effector memory T; TEMRA, effector memory RA T; Treg, regulatory T; DN, double negative; NKT, natural killer T; cTfh, circulating follicular helper T; expr, expression.

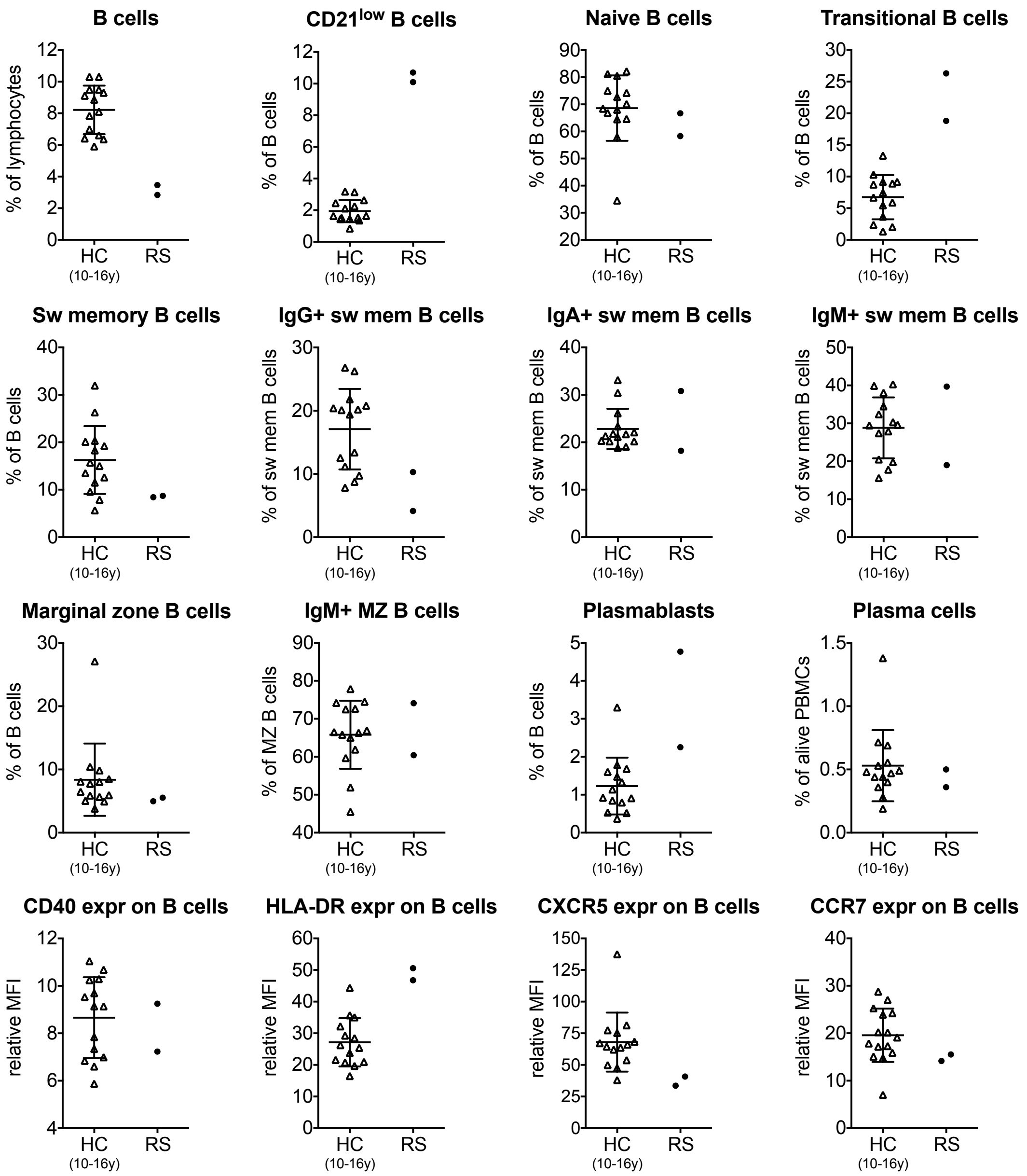

Supplementary Figure 3. Flow cytometric phenotyping of the family B patients: B cell subsets.
Flow cytometric immunophenotyping was performed on PBMCs of the 2 patients with *RNU4ATAC*-associated Roifman syndrome (RS) in comparison with age-matched healthy controls (HC). At time of analysis, the patients (II:1, II:2) were 14 and 11 years old, respectively. For details on gating, see reference 19. Relative mean fluorescence intensity (rMFI) was calculated by dividing the MFI of the positive population by the MFI of the Fluorescence Minus One (FMO) population. Graphs of HC group represent mean ± standard deviation. MZ, marginal zone; sw (mem), switched (memory); expr, expression.

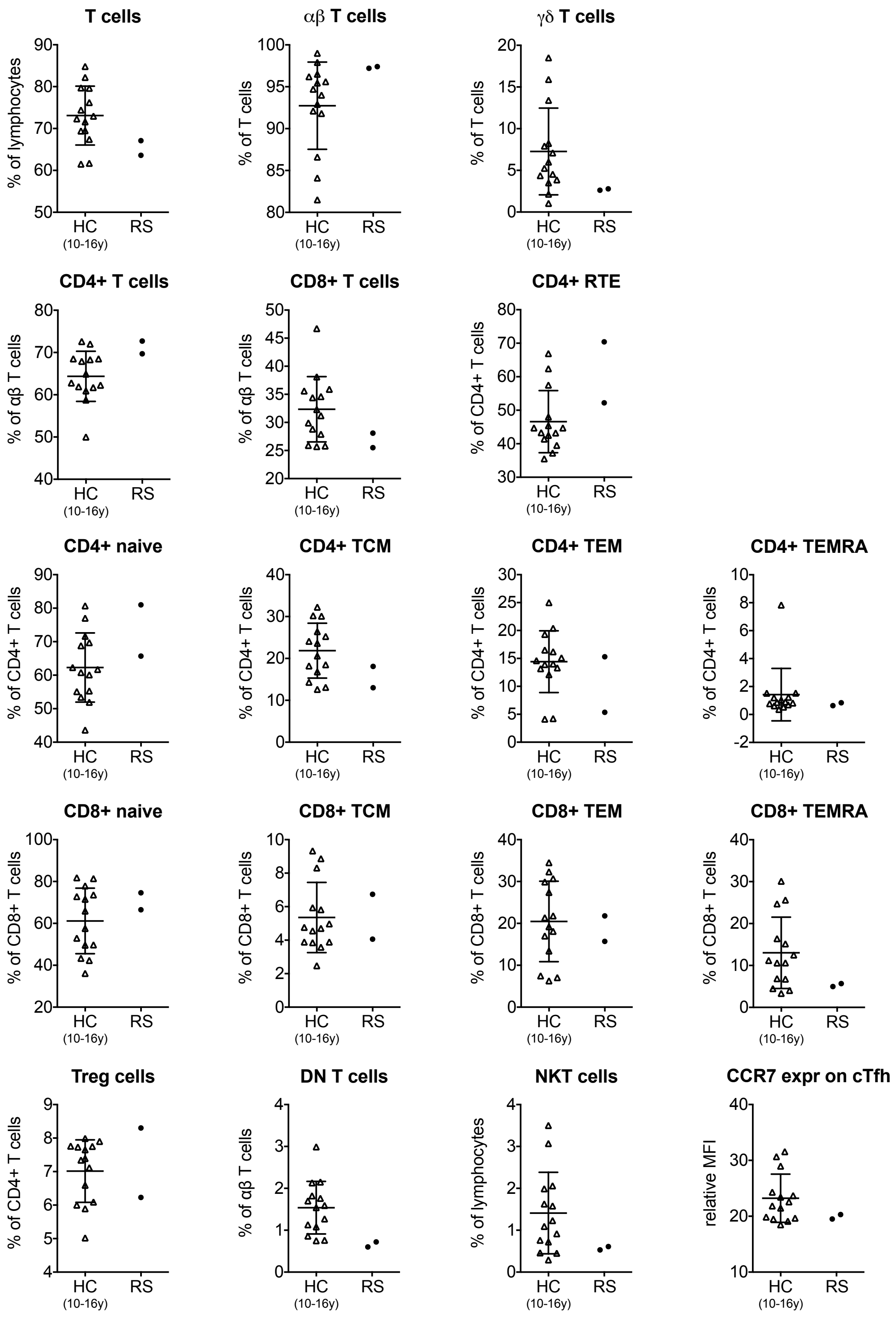

Supplementary Figure 4. Flow cytometric phenotyping of the family B patients: T cell subsets.
Flow cytometric immunophenotyping was performed on PBMCs of the 2 patients with RNU4ATAC-associated Roifman syndrome (RS) in comparison with age-matched healthy controls (HC). At time of analysis, the patients (II:1, II:2) were 14 and 11 years old, respectively. For details on gating, see reference 19. Relative mean fluorescence intensity (rMFI) was calculated by dividing the MFI of the positive population by the MFI of the Fluorescence Minus One (FMO) population. Graphs of HC group represent mean ± standard deviation. RTE, recent thymic emigrants; TCM, central memory T; TEM, effector memory T; TEMRA, effector memory RA T; Treg, regulatory T; DN, double negative; NKT, natural killer T; cTfh, circulating follicular helper T; expr, expression.
